# Ficolin-2 Plasma Level Assesses Liver Fibrosis in Non-Alcoholic Fatty Liver Disease

**DOI:** 10.3390/ijms23052813

**Published:** 2022-03-04

**Authors:** Pablo J. Giraudi, Noel Salvoza, Deborah Bonazza, Carlo Saitta, Daniele Lombardo, Biagio Casagranda, Nicolò de Manzini, Teresa Pollicino, Giovanni Raimondo, Claudio Tiribelli, Silvia Palmisano, Natalia Rosso

**Affiliations:** 1 Fondazione Italiana Fegato, Centro Studi Fegato, Area Science Park Basovizza Bldg.Q SS14 Km, 163.5, 34149 Trieste, Italy; noel.salvoza@fegato.it (N.S.); ctliver@fegato.it (C.T.); spalmisano@units.it (S.P.); natalia.rosso@fegato.it (N.R.); 2 Philippine Council for Health Research and Development, DOST Compound, Bicutan Taguig City 1631, Philippines; 3 Surgical Pathology Unit, Cattinara Hospital, ASUGI, 34149 Trieste, Italy; deborah.bonazza@asugi.sanita.fvg.it; 4 Department of Clinical and Experimental Medicine, Unit of Medicine and Hepatology, Laboratory of Molecular Hepatology, University Hospital of Messina, 98121 Messina, Italy; carlo.saitta@unime.it (C.S.); daniele.lombardo@unime.it (D.L.); giovanni.raimondo@unime.it (G.R.); 5 Surgical Clinic Division, Cattinara Hospital, ASUGI, 34149 Trieste, Italy; biagiocasa@gmail.com (B.C.); ndemanzini@units.it (N.d.M.); 6 Department of Medical, Surgical and Health Sciences, University of Trieste, 34149 Trieste, Italy; 7 Department of Human Pathology, Laboratory of Molecular Hepatology, University Hospital of Messina, 98121 Messina, Italy; teresa.pollicino@unime.it

**Keywords:** biomarkers, in silico, non-alcoholic fatty liver disease, discovery strategy, omics, blood-based tests, obesity, liver fibrosis

## Abstract

Fibrosis is the strongest predictor for disease-specific mortality in non-alcoholic fatty liver diseases (NAFLD), but the need for liver biopsy limits its diagnosis. We assessed the performance of plasma ficolin-2 (FCN-2) as a biomarker of fibrosis identified by an in silico discovery strategy. Two hundred and thirty-five morbidly obese (MO) subjects with biopsy-proven NAFLD stratified by fibrosis stage (F0, n = 44; F1, n = 134; F2, n = 46; F3/F4, n = 11) and 40 cirrhotic patients were enrolled. The cohort was subdivided into discovery (n = 76) and validation groups (n = 159). The plasma level of FCN-2 and other candidate markers was determined. FCN-2 was inversely correlated with the stage of liver fibrosis (ρ = −0.49, *p* < 0.001) independently of steatosis (*p* = 0.90), inflammation (*p* = 0.57), and ballooning (*p* = 0.59). In the global cohort, FCN-2 level decreased significantly in a stepwise fashion from F0/F1 (median 4753 ng/mL) to F2–F3–F4 (2760 ng/mL) and in cirrhotic subjects (1418 ng/mL). The diagnostic performance of FCN-2 in detecting F ≥ 2 was higher than other indexes (APRI, FIB-4) (AUROC 0.82, 0.68, and 0.6, respectively). The accuracy improved when combined with APRI score and HDL values (FCNscore, AUROC 0.85). Overall, the FCN-2 plasma level can accurately discriminate liver fibrosis status (minimal vs. moderate/advanced) significantly improving the fibrosis diagnostic algorithms.

## 1. Introduction

Non-alcoholic fatty liver disease (NAFLD) is the most common chronic liver disorder worldwide and is expected to become the leading cause of liver transplantation by 2030 [1,2]. Metabolic disorders such as type 2 diabetes mellitus (T2DM) and obesity increase the risk of developing severe liver disease in NAFLD [3].

NAFLD is divided into two clinical-histological entities: “simple steatosis” (NAFL) and “non-alcoholic steatohepatitis” (NASH). NASH is the progressive phenotype involving hepatocyte injury (ballooning), presence of inflammatory infiltrates, and fibrogenesis, with an elevated risk of cirrhosis and liver cancer [4]. Several reports have indicated fibrosis as the strongest predictor of long-term clinical outcomes in NAFLD patients [5].

Liver biopsy, the gold standard for NAFLD diagnosis, was established almost a century ago [6]. Despite the invasiveness, costs, and sampling error limitations, there are still no reliable non-invasive diagnostic tests for fibrosis in NAFLD. Two pathways have been exploited in approaching alternatives to the gold standard: blood-based non-invasive indirect tests have been combined in indices such as the fibrosis-4 (FIB-4) score [7], the AST to platelet ratio index (APRI), or individual markers in indirect tests such as the type III collagen neo-epitopes (PRO-C3) [8].

Imaging technologies (magnetic resonance elastography, shear wave elastography, or acoustic radial force imaging) have been used for the non-invasive diagnosis of NAFL/NASH and fibrosis. However, none of these modalities satisfies the desired clinical accuracy and practicability [9].

Interestingly, in the big-data era, new approaches contribute to the discovery of promising biomarkers. The fast development of omics technologies has enormously favored biomedical sciences. Specifically, the improvements in data acquisition and analysis through high-throughput technologies (such as microarrays and RNA-Seq) have the power to evolve biomedical science from a static to a more dynamic form [10]. Thus, the availability of harmonized datasets in many public repositories allows for in silico strategies to develop biomarker discovery pipelines.

Using enrichment analysis of phosphoproteomic datasets, Page et al. proposed C-C motif chemokine (CCL2) and tumor necrosis factor ligand superfamily member 6 (sFasL) as biomarkers in NAFLD pathogenesis [11]. Through a transcriptomic study, Hotta et al. [12] described core gene networks associated with NAFLD progression. Through transcriptomic meta-analysis, Ryaboshapkina et al. identified several genes involved in NAFLD progression and biomarkers for disease stratification [13].

In the present study, we assessed the performance of Ficolin-2 protein (FCN-2) as a putative novel biomarker of liver fibrosis and tested its utility combined in a blood-based score test. We also described the in silico strategy used to identify FCN-2 and other protein candidates that are potentially functional in fibrosis diagnosis.

## 2. Results

### 2.1. Identification by the In Silico Funnel Strategy of Candidate Biomarkers for Liver Fibrosis

**Figure 1:**
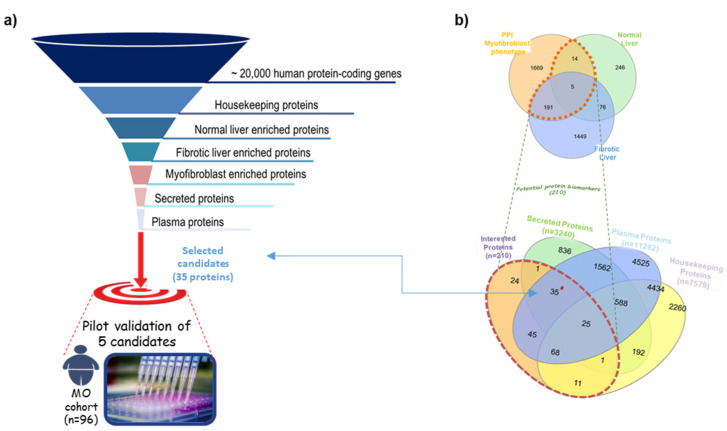
Summary of the in silico biomarker discovery strategy used in the study. (**a**) Layout of the in silico funnel strategy. (**b**) Venn diagrams illustrating the different datasets used to identify candidates satisfying our selection criteria.

We used a systematic discovery strategy in which the human proteome (originated from ~20,000 protein-coding genes [14]) was visualized at the top of a giant funnel. Several in silico filters were cross-placed at different heights (Figure 1). Proteins moving down in the funnel reach a thicker grade of selectivity and specificity. In the approach, the in silico filters are represented by Venn diagrams used to apply selection criteria at the bio-datasets, consequently obtaining the most selective sub-bio-datasets. The full description of the strategy is presented in Supplementary Materials—SM2. By using the described strategy, we identified some proteins as candidate biomarkers for liver fibrosis. These markers fulfilled the following desired characteristics: (1) candidates must participate in the acquisition of myofibroblast phenotype; (2) be expressed in liver tissue; (3) the expression must be modified during fibrosis; and (4) must be secreted and measurable in plasma. From this analysis, 35 proteins were identified as putative candidates, excluding collagen proteins or other extracellular matrix components, since they might be stabilized in the surrounding area of liver cells without the release into the bloodstream. Thus, we obtained 29 candidate biomarkers (Table 1). Five randomly selected proteins of the total candidates were evaluated in this study (Figure 1).

### 2.2. Demographics and Biochemical Data and Association with Liver Fibrosis

Table 2 summarizes the clinico-demographic details of the patients included in the discovery, validation, and combined MO cohorts. Since the primary endpoint of this study was to predict fibrosis, we stratified the cohorts according to the fibrosis stage (F0–F1 vs. F2–F3–F4). We used the discovery cohort to qualify the candidates and the validation cohort for their verification, testing the reproducibility of the diagnostic performances of the best candidate.

In Table 3, the clinico-demographic characteristics of the discovery cohort are presented. Significant differences between both fibrotic groups were observed in gender (30% female higher in the F0–F1 group) and blood parameters. AST and GGT levels were higher in the moderate/advanced fibrosis group (32 ± 18 IU/L vs. 23 ± 12 IU/L, *p* = 0.02, and 49 ± 41 IU/L vs. 32 ± 27 IU/L, *p* = 0.04, respectively) while blood platelets were reduced (220 ± 57 × 103/µL vs. 266 ± 60 × 103/µL, *p* = 0.002). The differences were also reflected by the blood-based indices such as FIB-4 (1.27 ± 1.1 F2/F3-F4 vs. 0.72 ± 0.3 F0–F1, *p* = 0.007), FORNS (4.2 ± 1.9 F2/F3–F4 vs. 3.0 ± 1.4 F0–F1, *p* = 0.003), and NFS (−0.06 ± 1.3 F2/F3–F4 vs. −1.2 ± 1.2 F0–F1, *p* = 0.0003).

The clinico-demographic characteristics of the validation cohorts are reported in SM1—Table S1. MO and cirrhotic cohorts showed significant differences in several parameters, particularly GGT, platelets, total cholesterol, and triglycerides, among others in the blood-based indices (APRI, FIB-4, NFS).

### 2.3. FCN-2 Plasma Levels Correlate with the Fibrosis Stage

**Figure 2:**
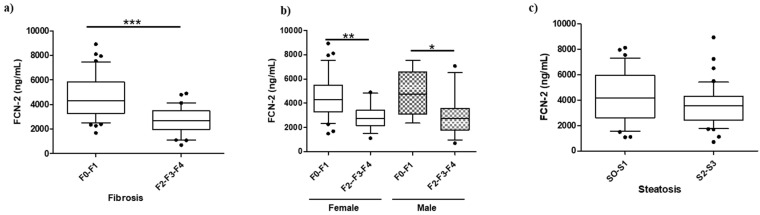
Boxplot for FCN-2 measurements in the morbidly obese discovery cohort. (**a**) Plasma abundances of FCN-2 determined by ELISA in MO subjects stratified by fibrosis stage, (**b**) MO stratified by gender and fibrosis stage, and (**c**) MO stratified by steatosis grade. *** Significant at *p* < 0.001, ** significant at *p* < 0.01, and * significant at *p* < 0.05.

The plasma level of the five candidates was assessed in all MO discovery samples (n = 76). No significant differences were found except for FCN-2 (SM1—Figure S1). The FCN-2 levels significantly decreased when liver fibrosis progressed from minimal to moderate/advanced stage (Figure 2a). FCN-2 was able to distinguish F0/F1 from F2/F3/F4 with levels decreasing from 4313 ng/mL (interquartile range: 3295–5849) to 2676 ng/mL (1983–3482), independently of gender and steatosis grade (Figure 2b,c).

Having shown an association between the plasma level of FCN-2 and the stage of fibrosis, we investigated the relationships with other biochemical and histological parameters. FCN-2 plasma level had a significant positive correlation with platelets (ρ = 0.37, *p* = 0.001) and a negative correlation with the fibrosis stage (ρ = −0.49, *p* < 0.001), FIB-4 (ρ = −0.32, *p* = 0.006), and NFS (ρ = −0.30, *p* = 0.01) (Table 4). The correlations indicate that changes in FCN-2 level reflect liver fibrosis but not steatosis, inflammation, and ballooning, as observed when the cohort was stratified by NAFLD stages or the grade of the different histological characteristics (SM1—Figures S2 and S3).

### 2.4. Diagnosis of Liver Fibrosis Using FCN Plasma Level

**Figure 3:**
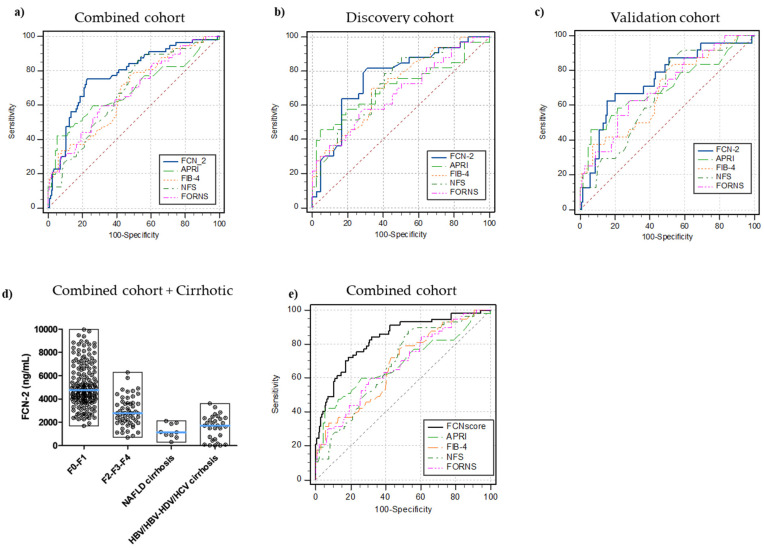
Receiver operating characteristic (ROC) curves for the diagnosis of significant fibrosis. FCN-2 vs. blood-based tests (APRI, FIB-4, NFS, FORNS) AUROCs in (**a**) combined cohort (n = 235, disease prevalence 24%), (**b**) discovery MO cohort (n = 76, disease prevalence 43%), (**c**) validation MO cohort (n = 159, disease prevalence 15%), (**d**) full picture for FCN-2 plasma levels in all samples included in the study, (**e**) FCNscore (FCN-2 levels, APRI, and HDL combined in a diagnostic model) vs. common blood-based tests (FIB-4, NFS, APRI, FORNS) AUROCs in the combined cohort. Significant fibrosis (F2, F3, and F4 stages).

In the discovery cohort (n = 76), an optimal FCN-2 cut-off level for the detection of moderate-advanced fibrosis was determined. FCN-2 of ≤3650 ng/mL had an AUROC of 0.79 for moderate-advanced fibrosis detection (sensitivity 85%, specificity 71%). This was replicated in the validation cohort (n = 159, AUROC = 0.80, sensitivity 71%, specificity 84%) and also in the overall combined cohort (n = 235, AUROC = 0.82, sensitivity 79%, specificity 81%) (Figure 3a–c). The FCN-2 plasma concentration in all analyzed samples stratified by liver injury is shown in Figure 3d.

Next, we compared the diagnostic performance of FCN-2 in detecting fibrosis with those from APRI, FIB-4, FORNS, and NFS indices. The FCN-2 marker had the best diagnostic accuracy for significant fibrosis diagnosis in the discovery, validation, and combined cohorts. Comparison of AUROCs using DeLong’s method demonstrated that FCN-2 was superior to APRI (*p* < 0.013), FIB-4 (*p* < 0.002), FORNS (*p* < 0.005), and NFS (*p* < 0.005). AUROCs, sensitivity, specificity, PPVs, NPVs, and significant comparisons for optimal cut-off values in the three cohorts are summarized in SM1—Table S2.

Several blood parameters or their combination in diagnostic scores displayed differences with the fibrosis stage. Using the data from the discovery cohort, logistic regression analysis was conducted to determine the appropriate variables to be included in the regression model, thus improving the diagnostic performance. AUROC of the combined model designed as FCNscore (FCN-2, APRI, and HDL) was 0.85 (95% confidence interval: 0.75 to 0.92) for the diagnosis of significant fibrosis (cut-off value > 0.35, specificity 86% and sensitivity 75%, Figure 3d) in the discovery cohort. In the validation and combined cohorts, AUROCs were 0.83 and 0.85, respectively. The number of variables included in the analysis and the estimated equation for the combined diagnostic model are detailed in SM1—Table S3. AUROC comparison analysis also demonstrated that FCNscore applied to the combined cohort was superior to all the included simple non-invasive scores: APRI, FIB-4, FORNS, and NFS with accuracies of 0.68 (*p* = 0.0001), 0.67 (*p* < 0.0001), 0.68 (*p* = 0.0001), and 0.68 (*p* = 0.0002), respectively (Supplementary Materials—Table S4). Using the FCNscore model in the combined cohort, the optimal threshold correctly staged 189 out of the 235 patients (80%) compared to 179 patients (76%) with APRI, 142 patients (60%) with FIB-4, 148 (63%) with FORNS, and 136 (58%) with NFS. Considering the negative predictive value (NPV) for each model, out of the 178 patients with non-significant fibrosis, 148 (83%) were staged correctly using FCNscore and 152 (85%), 100 (56%), 114 (64%), and 91 (51%) using APRI, FIB-4, FORNS, and NFS, respectively (Table 5).

## 3. Discussion

NAFLD affects a quarter of the global population and its prevalence is increasing in parallel with the increasing prevalence of obesity, MetS, and T2DM [1,15]. In obese populations, NAFLD prevalence varies from 60% to 95% [16]. Notably, NASH and fibrosis have been reported with prevalence ranges of 18–60 and 6–90% in severely obese subjects, respectively [17,18,19]. Not all NAFLD patients including those with NASH develop liver fibrosis. However, it is crucial to assess the fibrosis severity since it is one of the strongest predictors of liver-related complications and mortality [5].

The gold standard for diagnosing NAFLD and fibrosis stages is histological analysis. Considering the well-known limitations of liver biopsy (invasiveness, observer variability, sampling errors, among others), the development of alternatives is challenging in clinical management. In the present study, we assessed the plasma FCN-2 levels in a well-histologically characterized NAFLD obese cohort and evaluated its potential use for fibrosis diagnosis.

FCN-2 is among the 29 candidate biomarkers identified by a systematic in silico strategy. These candidates fulfil the desired selection criteria as relevant in the fibrotic process, secreted, and traceable in plasma. Specifically, 19 are differentially expressed in fibrotic liver and part of the myofibroblast phenotype acquisition PPI network (MyoPheNet); and nine are enriched proteins with elevated expression in healthy liver and MyoPheNet components. FCN-2 was the only candidate to accomplish the three main selection criteria: it is part of the MyoPheNet, shows reduced expression with fibrosis, and is expressed in healthy liver.

The main finding of this study is that the FCN-2 plasma level was strongly associated with the fibrosis stage assessed by histological analysis. Besides fibrosis, no association between histology and FCN-2 levels was shown, or if MO cohort was stratified into No NAFL, NAFL, and NASH. We found a reduction in FCN-2 level in MO subjects with significant fibrosis (≥2), and was further reduced in cirrhosis, regardless of fibrosis etiology. To our knowledge, this is the first study reporting on the potential use of plasma FCN-2 as a marker of fibrosis in morbidly obese patients. Our data agreed with those reported by Dai where FCN-2 and CPB2 proteins were shown as biomarkers of liver fibrosis through serum proteomics analysis and quantified using ELISA in a cohort of 46 CHB subjects [20]. Furthermore, Chen observed that intrahepatic expression and serum levels of FCN-2 were much lower in HCC and cirrhosis than in healthy controls [21]. In contrast, Liu reported an increase in serum FCN-2 associated with the severity of fibrosis and the activity of HCV infection [22].

FCN-2 is a serum protein expressed by hepatocytes and secreted into the circulation [23]. The serum/plasma median concentration in healthy people is approximately 5000 ng/mL; values below 1000 ng/mL have not been found in healthy adults. FCN2 plays a significant role in the host innate immunity. It appears to bind in human DNA and attaches to apoptotic/necrotic cells, thereby promoting their removal [24]. Relative FCN-2 deficiencies have been found to be associated with prematurity, low birth weight, and infections in neonates [25].

The plasma level of FCN-2 showed good discriminative power in categorizing hepatic fibrosis. The AUROC for diagnosis of significant fibrosis was 0.82 (F ≥ 2) and was superior to any other indices tested (FIB-4, FORNS, and NFS). Moreover, plasma FCN-2, when combined with APRI and HDL in FCN score, yielded an excellent discriminative power with an AUC of 0.85 and correctly identified 80% of patients included in the study.

The limitations of the study include relatively small cohort size and the low number of subjects with advanced fibrosis or cirrhosis. Nevertheless, our data strongly suggest that FCN-2 is a potential fibrosis biomarker and should be included in future non-invasive indices of hepatic fibrosis. Our observation needs to be validated in large independent cohorts such as the RESOLVE-IT [26] or the European NAFLD registry longitudinal cohort [27] in which candidates such as PRO-C3, YKL-40, A2M have recently been tested [8,22].

In conclusion, we developed an in silico strategy to detect putative proteins as biomarkers for fibrosis and demonstrated that FCN-2, either alone or in combination with APRI and HDL, is a good non-invasive diagnostic index for significant fibrosis

## 4. Materials and Methods

### 4.1. Study Design and Participants

The assessment of our candidate biomarkers was performed retrospectively in a cohort of morbidly obese (MO) subjects enrolled in a bariatric surgery program. The liver biopsy was performed at the time of the surgical procedure. All subjects provided their written consent to participate in the study. Sensitive data were protected through anonymization. The local ethical committee approved the study under protocol no. 22,979 (Comitato Etico Regionale Unico, FVG, SSN, Udine, Italy). Enrolled subjects were ≥18 years with a body mass index (BMI) > 40 kg/m^2^ (or >35 kg/m^2^ if obesity-related comorbidities were already present), with acceptable operative risks, failure of nonsurgical treatments, and declared compliance to follow lifelong medical surveillance. Subjects were excluded if they had coexistent chronic liver disease including suspected/confirmed hepatocellular carcinoma, alcoholic liver disease (>25 g/day alcohol consumption), known HBV, HCV, HIV positivity, and therapy with drugs that could affect the liver.

Blood samples from subjects with cirrhosis attributable to either NAFLD (n = 10) as well as chronic viral infection (HBV, HDV, and HCV) (n = 30) were collected and considered as positive controls of advanced fibrosis (F4).

### 4.2. Clinical Assessment

Anthropometric parameters including age, gender, body weight and height, BMI calculation, and waist circumference were recorded at the baseline visit. After overnight fasting, blood samples were collected before surgery to determine glucose, liver biochemistry (AST, ALT, GGT), albumin, platelets, lipid profile (TG, T-Chol, HDL), and others. Diabetes was diagnosed according to the ESC-EASD guidelines [28]. Blood-based tests of liver fibrosis such as FIB-4, APRI, FORNS, and NFS were calculated as described [29].

### 4.3. Liver Biopsy, Histopathology, Diagnosis of Fibrosis, and Cirrhosis

Liver specimens collected during the surgical procedure (wedge biopsy) were histologically analyzed by an expert pathologist blinded to all clinical data. Steatosis was graded according to the amount of fat (as lipid droplets in hepatocytes) on hematoxylin and eosin staining. Biopsies showing no or minimal (<5%) steatosis and absent injury or fibrosis were considered normal. The samples showing more than 5% steatosis were labelled as NAFLD. The histological diagnosis of NASH and fibrosis was made according to Kleiner–Brunt criteria [30]. In most cases, cirrhosis in positive controls was diagnosed by ultrasound and three via needle biopsy.

### 4.4. Assessment of Plasma Ficolin-2 and Other Candidates

Plasma levels of five of the total thirty-five candidates were measured by ELISA commercial kits: Fibrillin 1 (RayBio^®^ Human FBN1 ELISA Kit, E-EL-H2266, Elabscience, Houston, TX, USA), Insulin-like growth factor-binding protein 5 (RayBio^®^ Human IGFBP-5 ELISA Kit, ELH-IGFBP5, RayBiotech, Peachtree Corners, GA, USA), Noelin-2 (RayBio^®^ Human Olfactomedin 2, ELH-OLFM2, RayBiotech, Preachtree Corners, GA, USA), Urokinase-type plasminogen activator (Human U-Plasminogen Activator Simple Step ELISA^®^ Kit, ab226904, Abcam, Cambridge, UK), and Ficolin-2 (RayBio^®^ Human Ficolin-2 ELISA Kit, RayBiotech, Preachtree Corners, GA, USA).

### 4.5. Statistical Analysis

The MO cohort (n = 235) was divided into two subsequent cohorts: the discovery MO cohort including 76 MO subjects in which the prevalence of fibrosis was adjusted to 44% (enrichment of the moderate/advanced fibrosis proportion) and the validation MO cohort in which 159 MO subjects were included, maintaining the fibrosis prevalence (15%) close to those of the global MO population (24%). In both MO discovery and validation cohorts, the subjects were stratified according to fibrosis stage (F0–F1, minimal fibrosis; F2–F3–F4, moderate/advanced fibrosis) to assess the best candidate based on diagnostic performance analysis (accuracy and determination of optimal cut-off values). Continuous variables were expressed as mean ± standard deviation and categorical as numbers or percentages. Categorical variables were analyzed using chi-square tests with correction when appropriate. Independent t-test and ANOVA were used for normally distributed continuous variables. Non-parametric tests (Mann–Whitney, and Kruskal–Wallis) were applied for ordinal or continuous variables that failed to pass the D’Agostino and Pearson omnibus normality test. Correlation analyses were performed using Pearson or Spearman’s correlation coefficients to estimate the association of plasma levels and several factors of interest. Statistical analysis was performed using GraphPad Prism version 5.01 software. Logistic regression analysis was used to identify independent factors associated with fibrosis. The predictive model was built including the four best-associated variables (independent factors) selected after using the hierarchical forward selection algorithm in the subset selection test modality performed in NCSS statistical software (version 12.0.16). The diagnostic performance of the selected candidate FCN-2 was assessed by receiver operating characteristic (ROC) curves. The area under the ROC (AUROC) using DeLong method was used to compare the accuracy among the different fibrosis diagnostic tests. The sensitivity, specificity, positive predictive values (PPVs), and negative predictive values (NPVs) for relevant cut-offs (according to Youden’s index) were also calculated using MedCalc statistical software version 16.4.3.

## Figures and Tables

**Table 1:** List of the candidate biomarkers identified by the in silico funnel discovery strategy.

UniprotKb ID	Protein Name	Subcellular Location
Q15485	Ficolin-2	Secreted
P24593	Insulin-like growth factor-binding protein 5	Secreted
Q12805	EGF-containing fibulin-like extracellular matrix protein 1	Extracellular space
P27918	Properdin	Secreted
O75636	Ficolin-3	Secreted
Q8NEA6	Zinc finger protein GLIS3	Nucleous, predicted secreted
O95897	Noelin-2	Secreted
P13726	Keratin, type I cytoskeletal 10	Extracellular region
Q13332	Receptor-type tyrosine-protein phosphatase S	Cell membrane, secreted
Q9H6X2	Anthrax toxin receptor 1	Cell membrane, secreted
Q00796	Sorbitol dehydrogenase	Mitochondrion
P35555	Fibrillin 1	Secreted, extracellular matrix
Q9Y6N6	Laminin subunit gamma-3	Extracellular matrix, secreted
P09341	Growth-regulated alpha protein	Secreted
P24592	Insulin-like growth factor-binding protein 6	Secreted
P00749	Urokinase-type plasminogen activator	Secreted
Q9UKQ2	Disintegrin and metalloproteinase domain-containing protein 28	Secreted
P13612	Integrin alpha-4	Membrane, secreted
P21246	Pleiotrophin	Secreted
Q9UM22	Mammalian ependymin-related protein 1	Secreted
P05156	Complement factor I	Extracellular space
P03950	Angiogenin	Nucleous, secreted
P19652	Alpha-1-acid glycoprotein 2	Secreted
P04114	Apolipoprotein B-100	Secreted
P00747	Plasminogen	Secreted
Q14624	Inter-alpha-trypsin inhibitor heavy chain H4	Secreted
P02749	Beta-2-glycoprotein 1	Secreted
P09237	Matrilysin	Secreted
P81172	Hepcidin	Secreted

Full list of the putative candidates identified by the in silico funnel strategy. UniprotKb ID were the identifiers used to follow the proteins during the selection process. Protein full names and localization are those reported in the UniProt database. The selection criteria fulfilled by each candidate are also reported.

**Table 2:** Clinical characteristics of all morbidly obese patients.

Variable	Combined Cohort (n = 235)	Discovery Cohort (n = 76)	Validation Cohort (n = 159)	*p* Value
Age (years)	45.0 ± 10.0	44.5 ± 10.6	45.3 ± 9.8	0.95
Gender (female)	159 (67%)	52 (68%)	107 (67%)	0.98
BMI (Kg/m^2^)	43.9 ± 5.8	43.9 ± 6.4	43.9 ± 5.5	0.96
Fasting glucose (mg/dL)	112.6 ± 30.0	118.2 ± 33.4	110 ± 28.0	0.27
T2DM (yes)	61 (26%)	19 (25%)	42 (26%)	0.97
AST (U/L)	25.9 ± 14.0	26.8 ± 15.2	25.8 ± 14.0	0.93
ALT (U/L)	32.4 ± 24.6	34.9 ± 29.6	31.2 ± 22.0	0.79
GGT (U/L)	39.7 ± 39.2	39.1 ± 34.6	40.0 ± 41.3	0.81
Albumin (g/dL)	4.2 ± 0.3	4.2 ± 0.3	4.1 ± 0.3	0.06
Platelets (×10^9^/L)	251.0 ± 68.0	253.0 ± 71.0	246.0 ± 63.0	0.68
Total Cholesterol (mg/dL)	205.0 ± 41.0	200.0 ± 49.0	207 ± 37.5	0.45
HDL cholesterol (mg/dL)	47.3 ± 10.7	48.1 ± 10.0	45.7 ± 12.0	0.08
Triglycerides (mg/dL)	144.0 ± 77.0	140.0 ± 67.3	151.5 ± 93.2	0.70
Steatosis grade (0/1/2/3)	53/79/61/42	12/22/27/15	41/57/34/27	0.02 *
Lobular inflammation (0/1/2/3)	76/134/25/0	23/39/14/0	53/95/11/0	0.18
Ballooning (0/1/2)	124/68/43	31/28/17	93/40/26	0.16
Fibrosis stage (0/1/2/3ߝ4)	45/132/46/12	18/24/28/6	27/108/18/6	<0.0001 ***
AST/ALT	0.92 ± 0.31	0.93 ± 0.30	0.90 ± 0.32	0.67
APRI	0.32 ± 0.57	0.32 ± 0.68	0.30 ± 0.24	0.98
FIB4	0.94 ± 0.68	0.95 ± 0.79	0.93 ± 0.61	0.88
FORNS	3.57 ± 1.8	3.55 ± 1.73	3.56 ± 1.80	0.95
NFS	−0.93 ± 1.36	−0.88 ± 1.35	−0.70 ± 1.38	0.57

Data are shown as mean ± SD for continuous variables, number (%) for binary variables, and frequency for categorical variables. ANOVA was used to test for significant differences within continuous variables that were normally distributed while the Kruskal–Wallis with Dunn post-test was used when not normally distributed. Chi-square test was used for categorical variables. *** Significant at *p* < 0.001, and * significant at *p* < 0.05. Abbreviations: BMI, body mass index; ALT, alanine aminotransferase; AST, aspartate aminotransferase; GGT, gamma-glutamyl transferase; T2DM, type 2 diabetes mellitus; HDL, high density cholesterol; APRI, AST to platelet ratio index; FIB4, fibrosis-4; FORNS, Forns index; NFS, NAFLD fibrosis score.

**Table 3:** Demographic and clinical characteristics of the discovery cohort.

MO Discovery Cohort			
Variable	F0–F1 (Minimal Fibrosis) n = 42	F2–F3 (Moderate/Advance Fibrosis) n = 34	*p* Value
Age (years)	45.0 ± 10.0	44.5 ± 10.6	0.27
Gender (female)	35 (81%)	17 (51%)	0.005 **
BMI (kg/m^2^)	42.8 ± 10.4	45.4 ± 7.1	0.09
Fasting glucose (mg/dL)	115.1 ± 33.5	122.2 ± 33.4	0.37
T2DM (yes)	8 (19%)	11 (33%)	0.16
AST (UI/L)	23.1 ± 11.7	32.0 ± 18.0	0.02 *
ALT (UI/L)	32.6 ± 29.5	38.0 ± 29.9	0.44
GGT (UI/L)	31.8 ± 27.5	48.6 ± 40.6	0.04
Albumin (g/dL)	4.1 ± 0.3	4.2 ± 0.4	0.09
Platelets (X10^9^/L)	266 ± 60	220 ± 57	0.002 **
Total Cholesterol (mg/dL)	201.7 ± 47.3	198.5 ± 52.0	0.78
HDL Cholesterol (mg/dL)	47.4 ± 13.3	43.6 ± 10.0	0.18
Triglycerides (mg/dL)	150.8 ± 108	152.4 ± 70.0	0.94
Steatosis grade (0, 1, 2, 3)	6/15/14/7	6/7/13/8	0.55
Lobular Inflammation (0, 1, 2, 3)	17/19/6/0	6/20/8/0	0.09
Ballooning (0, 1, 2)	17/16/9	14/12/8	0.91
Fibrosis stage (0, 1, 2, 3, 4)	18/24/0/0/0	0/0/28/5/1	<0.0001 ***
AST/ALT	0.87 ± 0.33	0.94 ± 0.32	0.33
APRI	0.22 ± 0.12	0.41 ± 0.35	0.002 **
FIB-4	0.72 ± 0.30	1.27 ± 1.09	0.007 **
FORNS	3.0 ± 1.4	4.24 ± 1.9	0.003 **
NFS	−1.2 ± 1.2	−0.06 ± 1.3	0.0003 ***

Data are shown as mean ± SD for continuous variables, number (%) for binary variables, and frequency for categorical variables. ANOVA was used to test for significant differences within continuous variables that were normally distributed while the Kruskal–Wallis with Dunn post-test was used when not normally distributed. Chi-square test was used for categorical variables. *** Significant at *p* < 0.001, ** significant at *p* < 0.01, and * significant at *p* < 0.05. Abbreviations: BMI, body mass index; ALT, alanine aminotransferase; AST, aspartate aminotransferase; GGT, gamma-glutamyl transferase; T2DM, type 2 diabetes mellitus; HDL, high density cholesterol; APRI, AST to platelet ratio index; FIB4, fibrosis-4; FORNS, Forns index; NFS, NAFLD fibrosis score; NA, not available.

**Table 4:** Correlations between the blood parameters and fibrosis in the discovery cohort.

XY, n = 76	X = [FCN-2]		X = Fibrosis Kleiner Score	
Parameter (Y)	Rho	*p* Value	Rho	*p* Value
BMI	−0.071	0.54	0.25	0.03
Triglycerides	0.23	0.05	0.23	0.06
Total Cholesterol	0.064	0.68	−0.1	0.39
Glc	0.024	0.83	0.2	0.09
AST	−0.056	0.62	0.32	0.006
ALT	0.035	0.77	0.22	0.06
GGT	0.026	0.82	0.39	0.004
Platelets	0.37	0.001	−0.33	0.004
INR	−0.3	0.009	0.33	0.004
Albumin	−0.047	0.68	0.17	0.14
AST/ALT ratio	−0.12	0.3	0.08	0.5
Steatosis score	−0.014	0.901	0.13	0.25
Lob. Inflammation score	−0.06	0.575	0.33	0.004
Portal Inflammation score	−0.028	0.811	0.25	0.03
Ballooning score	−0.062	0.593	0.11	0.34
Fibrosis score	−0.49	<0.001	-	-
APRI	−0.15	0.19	0.37	<0.001
FIB-4	−0.32	0.006	0.4	<0.001
FORNS	−0.22	0.06	0.36	0.002
NFS	−0.3	0.01	0.45	<0.001

Histological scores according to Kleiner–Brunt classification. Pearson’s or Spearman’s correlation coefficient (Rho) measures the strength and direction of association between the two variables under study. Abbreviations: BMI, body mass index; ALT, alanine aminotransferase; AST, aspartate aminotransferase; GGT, gamma-glutamyl transferase; T2DM, type 2 diabetes mellitus; HDL, high density cholesterol; APRI, AST to platelet ratio index; FIB4, fibrosis-4; FORNS, Forns index; NFS, NAFLD fibrosis score.

**Table 5:** Classification of subjects in the combined cohort according to moderate/advanced fibrosis (prevalence 25%, n = 235).

	F0–F1, n = 178 (“Rule Out” Significant Fibrosis)		F2–F3–F4, n = 57 (“Rule in” Significant Fibrosis)		Total, N = 235	
	Correctly Identified	Incorrectly Identified	Correctly Identified	Incorrectly Identified	Well-Classified	Misclassified
**Parameter/model**	n/N (%)	n/N (%)	n/N (%)	n/N (%)	n/N (%)	n/N (%)
FCNscore (>0.344)	148/178 (83%)	30/178 (17%)	41/57 (72%)	16/57 (28%)	189/235 (80%)	46/235 (20%)
FCN-2 (<3650)	144/178 (81%)	34/178 (19%)	45/57 (79%)	12/57 (21%)	189/235 (80%)	46/235 (20%)
APRI (>0.35)	152/178 (85%)	26/178 (15%)	27/57 (47%)	30/57 (53%)	179/235 (76%)	43/235 (24%)
FIB-4 (>0.78)	100/178 (56%)	78/178 (44%)	42/57 (74%)	15/57 (26%)	142/235 (60%)	93/235 (40%)
FORNS (>3.72)	114/178 (64%)	64/178 (46%)	34/57 (60%)	23/57 (40%)	148/235 (63%)	82/235 (35%)
NFS (>-0.96)	91/178 (51%)	87/178 (49%)	45/57 (79%)	12/57 (21%)	136/235 (58%)	99/235 (42%)

## Data Availability

Not applicable.

## Supplementary Materials

The supporting information SM1 and SM2 can be downloaded at: https://www.mdpi.com/article/10.3390/ijms23052813/s1.
