# Supplementary material for: Ficolin-2 Plasma Level Assesses Liver Fibrosis in Non-Alcoholic Fatty Liver Disease
Source: Int J Mol Sci. 2022 Mar 4;23(5):2813. doi: 10.3390/ijms23052813 (PMC8911336; doi:10.3390/ijms23052813)
Supplement: Supplementary file 1 [file ijms-23-02813-s001.zip › ijms-1619225-supplementary1.pdf]

Supplementary Material 1 (SM1)

Ficolin-2 Plasma Levels Assesses Liver Fibrosis in Non-Alcoholic Fatty Liver Disease

Pablo J. Giraudi [1,*], Noel Salvoza [1,2], Deborah Bonazza [3], Carlo Saitta [4], Daniele Lombardo [4], Biagio Casagranda [5], Nicolò de Manzini [5,6], Teresa Pollicino [7], Giovanni Raimondo [4], Claudio Tiribelli [1], Silvia Palmisano [1,5,6] and Natalia Rosso [1]

[1] Fondazione Italiana Fegato, Centro Studi Fegato, Area Science Park Basovizza Bldg.Q SS14 Km,163.5, 34149 Trieste, Italy; noel.salvoza@fegato.it (N.S.); ctliver@fegato.it (C.T.); spalmisano@units.it (S.P.); natalia.rosso@fegato.it (N.R.)
[2] Philippine Council for Health Research and Development, DOST Compound, Bicutan Taguig City 1631, Philippines
[3] Surgical Pathology Unit, Cattinara Hospital, ASUGI, 34149 Trieste, Italy; deborah.bonazza@asugi.sanita.fvg.it
[4] Department of Clinical and Experimental Medicine, Unit of Medicine and Hepatology, Laboratory of Molecular Hepatology, University Hospital of Messina, 98121 Messina, Italy; carlo.saitta@unime.it (C.S.); daniele.lombardo@unime.it (D.L.); giovanni.raimondo@unime.it (G.R.)
[5] Surgical Clinic Division, Cattinara Hospital, ASUGI, 34149 Trieste, Italy; biagiocasa@gmail.com (B.C.); ndemanzini@units.it (N.d.M.)
[6] Department of Medical, Surgical and Health Sciences, University of Trieste, 34149 Trieste, Italy
[7] Department of Human Pathology, Laboratory of Molecular Hepatology, University Hospital of Messina, 98121 Messina, Italy; teresa.pollicino@unime.it
* Correspondence: pablo.giraudi@fegato.it; Tel.: +39-040-375-7923; Fax: +39-040-375-7832

Number of supplementary tables: 4

Number of supplementary figures: 3

Table S1. Clinical characteristics of the validation cohorts.

	MO Validation cohort (n=159)		Cirrhotic cohort (n=40)	p value	
**Variable**	**F0-F1, n=135**	**F2-F3, n=24**	**Positive controls**	**F2-F3 vs F0-F1**	**Cirrhotic vs F0-F1, F2-F3**
**Age (years)**	45 ± 10	47 ± 10	68 ± 12	0.3	<0.001, <0.001
**Gender (female)**	95 (70%)	11 (46%)	16 (40%)	0.02	<0.001, 0.80
**BMI (kg/m²)**	44 ± 5	44 ± 6	27 ± 3	0.98	<0.001, <0.001
**Fasting glucose (mg/dL)**	108 ± 27	121 ± 32	122 ± 44	0.14	0.02, 0.72
**T2DM (yes)**	33 (24%)	9 (37%)	20 (50%)	0.12	0.003, 0.78
**AST (UI/L)**	24 ± 12	34 ± 17	28 ± 13	0.01	0.02, 0.32
**ALT (UI/L)**	30 ± 21	40 ± 24	24 ± 10	0.04	0.39, 0.02
**GGT (UI/L)**	36 ± 39	61 ± 48	48 ± 33	0.01	0.02, 0.60
**Albumin (g/dL)**	4.2 ± 0.3	4.2 ± 0.3	3.9 ± 0.5	0.32	<0.001, 0.002
**Platelets (X10⁹/L)**	259 ± 71	217 ± 57	101 ± 60	0.007	<0.001, <0.001
**Total Cholesterol (mg/dL)**	209 ± 36	193 ± 36	149 ± 37	0.06	<0.001, 0.002
**HDL cholesterol (mg/dL)**	49 ± 10	44 ± 10	50 ± 14	0.03	0.6, 0.07
**Triglycerides (mg/dL)**	138 ± 66	153 ± 73	104 ± 33	0.28	0.004, 0.004
**Steatosis grade (0. 1. 2. 3)**	34/55/24/22	7/2/10/5	NA	0.008	NA
**Lobular Inflammation (0. 1. 2. 3)**	47/82/6/0	6/13/5/0	NA	0.014	NA
**Ballooning (0. 1. 2)**	80/34/21	13/6/5	NA	0.62	NA
**Fibrosis stage (0. 1. 2. 3. 4)**	27/108/0/0	0/0/18/6	0/0/0/0/40	<0.001	NA
**AST/ALT**	0.9 ± 0.3	0.9 ± 0.3	1.3 ± 0.5	0.6	<0.001, 0.005
**APRI**	0.3 ± 0.7	0.4 ± 0.3	0.9 ± 0.8	0.002	<0.001, <0.001
**FIB-4**	0.8 ± 0.3	1.4 ± 1.2	5.1 ± 3.3	0.006	<0.001, <0.001
**NFS**	.-0.8 ± 1.3	.-0.1 ± 1.4	0.7 ± 1.4	0.01	<0.001, 0.01

Morbidly obese (MO) and Cirrhotic validation cohorts. Data are shown as mean ± SD for continuous variables, number (%) for binary variables, and frequency for categorical variables. ANOVA was used to test for significant differences within continuous variables that were normally distributed while Kruskal-Wallis with Dunn post-test when not normally distributed. Chi-Square test was used for categorical variables. significant at $p < 0.05$. Abbreviations: BMI, body mass index; ALT, alanine aminotransferase; AST, aspartate aminotransferase; GGT, gamma-glutamyl transferase; T2DM, type 2 diabetes mellitus; HDL, high density cholesterol; APRI, AST to platelet ratio index; FIB4, fibrosis-4; FORNS, Forns index; NFS, NAFLD fibrosis score.

Table S2. Comparison of the performance of each test for the diagnosis of significant fibrosis in the MO cohorts.

Biomarker/Test	AUROC (95% CI)	Sens (%)	Spec (%)	PPV	NPV	P value
**Discovery MO cohort (n = 76, prevalence 44%)**						
FCN-2 ≤ 3650	0.79 (0.68-0.88)	85	71	70	86	-
APRI > 0. 35	0.71 (0.59-0.81)	45	95	88	69	0.27
FIB-4 > 0.78	0.71 (0.60-0.81)	70	67	62	74	0.28
FORNS > 3.72	0.67 (0.55-0-78)	57	71	61	68	0.13
NFS > -0.96	0.73 (0.61-0.82)	79	59	60	78	0.40
**Validation MO cohort (n = 159, prevalence 15%)**						
FCN-2 ≤ 3650	0.80 (0.73-0.86)	71	84	44	94	-
APRI > 0. 35	0.70 (0.62-0.77)	50	80	36	90	0.23
FIB-4 > 0.78	0.68 (0.66-0.75)	75	54	22	92	0.09
FORNS > 3.72	0.70 (0.62-0.77)	62	71	28	91	0.16
NFS > -0.96	0.66 (0.57-0.73)	71	51	21	90	0.05
**Combined MO cohort (n = 235, prevalence 24%)**						
FCN-2 ≤ 3650	0.82 (0.76-0.87)	79	81	58	92	-
APRI > 0. 35	0.68 (0.62-0.74)	47	87	55	83	0.013
FIB-4 > 0.78	0.67 (0.61-0.73)	72	57	36	86	0.002
FORNS > 3.72	0.68 (0.61-0.73)	59	68	38	84	0.005
NFS > -0.96	0.68 (0.61-0.74)	79	53	36	88	0.005

AUROC. Sens. sensitivity; Spec. specificity; PPV. positive predictive value; NPV. negative predictive value. Cut-off according to Youden index J.

Table S3. Subset selection summary of FCNscore diagnostic equation model.

Step	Action	N° of terms	Log-likelihood	R^2 value	R^2 change	Term entered
1	Add	1	-128	0.00	0.00	Intercept
2	Add	2	-101	0.21	0.21	FCN-2
3	Add	3	-91	0.29	0.07	APRI
4	Add	4	-89	0.30	0.0	HDL

Model for Logit(Fib_diagnosis) = XB when Fib_diagnosis = 1

FCNscore model: 3.567 + 4.183*APRI -0.0007*FCN_2 -0.039*HDL

The model was obtained applying the subset selection option in logistic regression analysis at data from the discovery cohort. The hierarchical forward algorithm was used to manage the term's interactions. Y's reference value was set as 0. obtaining the regression model for the outcome of our interest (value of 1. significant fibrosis diagnosis). The total number of independent variables included in the analysis was 17 (numerical variables: Age. BMI. AST. ALT. GGT. fasting Glc. total cholesterol. HDL. TG. PLT. albumin. AST/ALT. FIB-4. FORNS. APRI. NFS and FCN-2; categorical variables: sex. gender and diabetes) and the maximum number of iterations was fixed in 20. According to log-likelihood values. the test report showed that four terms (the intercept and the three variables – FCN-2. APRI. and HDL-) provide the best model. The addition of the fifth variable did not increase the R-squared value very much.

Table S4. Diagnostic accuracies for FCNscore and blood-based indexes in the combined MO cohort.

**Combined MO Cohort (n = 235, prevalence 25%)**						
**Biomarker/Test**	**AUROC (95% CI)**	**Sens (%)**	**Spec (%)**	**PPV**	**NPV**	**P value**
FCNscore > 0.35	0.85 (0.79-0.89)	72	84	60	90	-
FCN-2 ≤ 3650	0.82 (0.76-0.87)	79	81	58	92	0.14
APRI > 0. 35	0.68 (0.62-0.74)	47	87	55	83	0.0001
FIB-4 > 0.78	0.67 (0.61-0.73)	72	57	36	86	<0.0001
FORNS > 3.72	0.68 (0.61-0.73)	59	68	38	83	0.0001
NFS > -0.96	0.68 (0.61-0.74)	79	53	36	88	0.0002

Pairwise comparison of AUROCs was according to DeLong's method, considering significant $p < 0.05$ (FCNscore *vs* other models or markers).

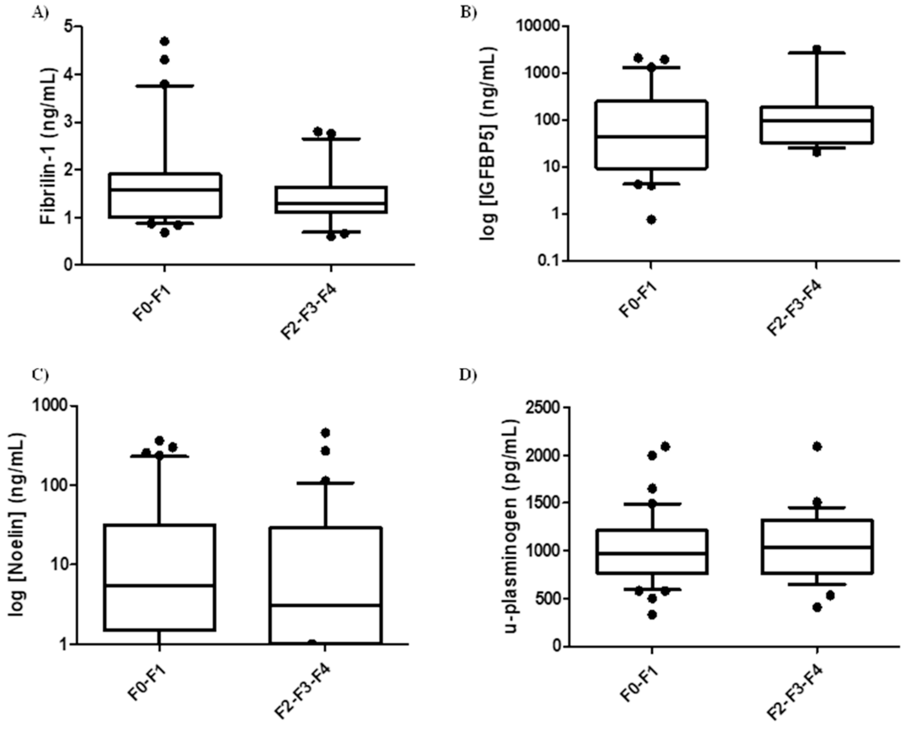

Figure S1. Plasma abundances of 4 candidates in the MO cohort stratified by fibrosis stage.The plasma level of candidates was assessed by ELISA kits. **A)** Fibrillin-1, **B)** IGFBP5, **C)** Noelin, and **D)** U-plasminogen. F0-F1 (non significant/minimal fibrosis) and F2-F3-F4 (significant/moderated-advanced fibrosis) in the MO cohort. Values of concentration were expressed in the log scale for Noelin and IGFBP5 for better visualization.

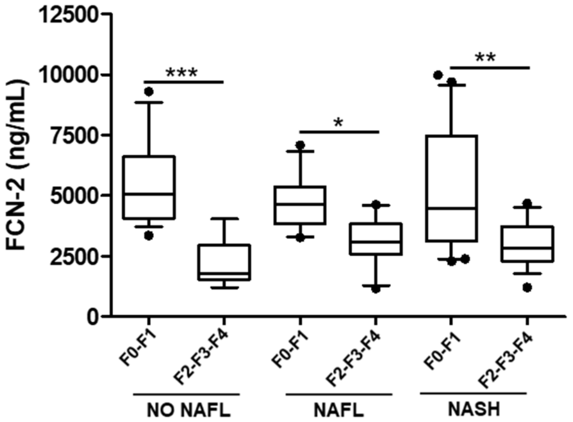

Figure S2. Bloxpot of FCN-2 plasma levels in the MO discovery cohort stratified by liver histology. Plasma samples from 76 MO patients were stratified by fibrosis and NAFLD stage. NO NAFL (F0-F1, n=6; F2-F3-F4, n=6); NAFL (F0-F1, n=13; F2-F3-F4, n=11) and NASH (F0-F1, n=23; F2-F3-F4, n=17). ***significant at $p < 0.001$, **significant at $p < 0.01$, and *significant at $p < 0.05$.

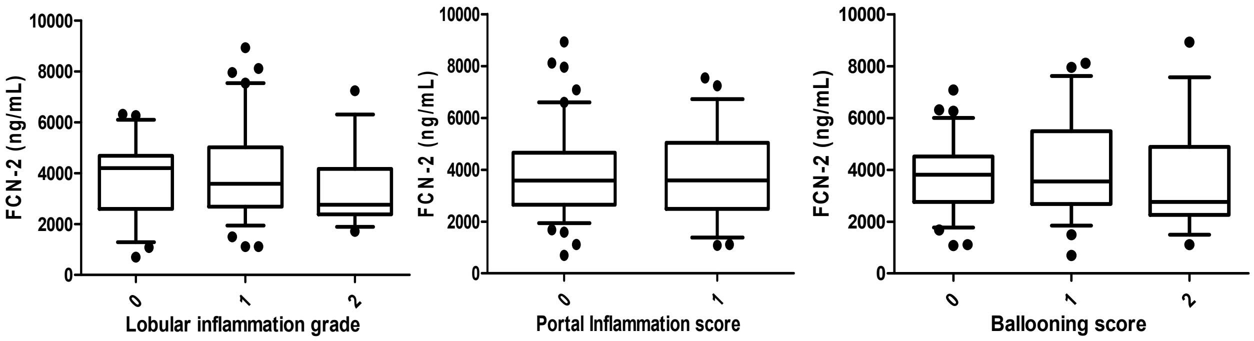

Figure S3. FCN-2 plasma level in the MO cohort stratified by liver histology. **A)** lobular inflammation, **B)** ballooning, and **C)** portal inflammation. Histological scores were based on Kleiner-Brunt classification.
