# Supplementary material for: Ficolin-2 Plasma Level Assesses Liver Fibrosis in Non-Alcoholic Fatty Liver Disease
Source: Int J Mol Sci. 2022 Mar 4;23(5):2813. doi: 10.3390/ijms23052813 (PMC8911336; doi:10.3390/ijms23052813)
Supplement: Supplementary file 1 [file ijms-23-02813-s001.zip › ijms-1619225-supplementary2.pdf]

Supplementary Material 2 (SM2)

Ficolin-2 Plasma Levels Assesses Liver Fibrosis in Non-Alcoholic Fatty Liver Disease

Pablo J. Giraudi [1,*], Noel Salvoza [1,2], Deborah Bonazza [3], Carlo Saitta [4], Daniele Lombardo [4], Biagio Casagranda [5], Nicolò de Manzini [5,6], Teresa Pollicino [7], Giovanni Raimondo [4], Claudio Tiribelli [1], Silvia Palmisano [1,5,6] and Natalia Rosso [1]

[1] Fondazione Italiana Fegato, Centro Studi Fegato, Area Science Park Basovizza Bldg.Q SS14 Km,163.5, 34149 Trieste, Italy; noel.salvoza@fegato.it (N.S.); ctliver@fegato.it (C.T.); spalmisano@units.it (S.P.); natalia.rosso@fegato.it (N.R.)
[2] Philippine Council for Health Research and Development, DOST Compound, Bicutan Taguig City 1631, Philippines
[3] Surgical Pathology Unit, Cattinara Hospital, ASUGI, 34149 Trieste, Italy; deborah.bonazza@asugi.sanita.fvg.it
[4] Department of Clinical and Experimental Medicine, Unit of Medicine and Hepatology, Laboratory of Molecular Hepatology, University Hospital of Messina, 98121 Messina, Italy; carlo.saitta@unime.it (C.S.); daniele.lombardo@unime.it (D.L.); giovanni.raimondo@unime.it (G.R.)
[5] Surgical Clinic Division, Cattinara Hospital, ASUGI, 34149 Trieste, Italy; biagiocasa@gmail.com (B.C.); ndemanzini@units.it (N.d.M.)
[6] Department of Medical, Surgical and Health Sciences, University of Trieste, 34149 Trieste, Italy
[7] Department of Human Pathology, Laboratory of Molecular Hepatology, University Hospital of Messina, 98121 Messina, Italy; teresa.pollicino@unime.it
* Correspondence: pablo.giraudi@fegato.it; Tel.: +39-040-375-7923; Fax: +39-040-375-7832

Number of sections: 3

Number of supplementary tables: 13 excel tables

Number of supplementary figures: 4

Electronic word count: 1493 (without References)

1. Methods

1.1 Data collection and processing

To develop our strategy, we collected datasets from Human Protein Atlas (HPA) [1], Genome-based tissue expression consortium (GTEx) [2], medical literature by searching in PubMed (query terms: transcriptomics in activated human hepatic stellate cells / human myofibroblasts) [3]

[4] [5], Plasma Proteome Database [6] and Plasma PeptideAtlas [7], between other data resources (Table 1A and 2A).

At the time in which the analysis was performed, limited information on transcriptomics data was available, we retrieved only one original study about human myofibroblasts [4] and another on liver fibrosis in patients with NASH [3]. The review of Uhlén M. et al. was selected due to the extensive revision on studies containing transcriptome datasets for different healthy human tissues available online [8].

In our systematic strategy, the identifiers for protein-coding genes in the consulted data resources were standardized, through mapping to the UniProtKB identifiers on UniProt database [9], using UniProt retrieve/ID mapping tool or PICR tool from EBI-EMBL [10]. We used UniProtKB IDs because they are stable; they do not change or update once assigned. Moreover, generally, they reflect isoforms information and are recognized by many public protein data repositories.

Dataset comparison and sub-groups selection was performed by applying Venn diagrams using InteractiVenn web-based tool [13]. Venn diagrams were used as *in silico filters* to identify the interested proteins, those fulfilling the following desired criteria. They should be fibrotic liver-specific, predictive to be secreted, experimental evidenced in plasma, and not included as part of the housekeeping proteome.

Protein-protein interaction biological network was generated by using Cytoscape software 3.6.0, acquiring only interaction data from databases complying with the International Molecular Exchange Consortium (IMEx) curation rules [11]. Reactome pathway knowledgebase Version 65 was used to analyze the selected candidate biomarkers' searching for over-represented biological pathways [12].

2. Results

2.1 Data collection and identification of protein-encoding genes involved in the acquisition of myofibroblasts phenotype by hepatic stellate cells

As is well known, when the liver is chronically injured, myofibroblasts originate predominantly from activated hepatic stellate cells (HSCs), become the leading players in extracellular matrix remodeling, and enable the fibrogenic process and its perpetuation [13] [14]. The cellular transdifferentiation process that gradually occurs *in vivo* can be mimicked *in vitro* (by the short-term culture of HSC on plastic plates), allowing, the study of biomolecular events regulating cell activation/transdifferentiation [4]. Boers W. and colleagues, performed that sort of task, analyzing the gene expression profile in fully transdifferentiated hepatic myofibroblasts, and demonstrated that 43 genes were exclusively expressed in this phenotype. Moreover, they also evidenced that other 16 genes showed high expression in myofibroblasts, low in activated HSC, and were not expressed when HSC were freshly isolated.

In this study, we matched the 59 genes to their protein UniProt identifiers (full description of the dataset in Table 1A, Table 3A, and Figure 1A-a). Then, to overcome the low probability of finding useful biomarkers from the low starting number of candidates, we decided to build a protein-protein interaction (PPI) biological network between those 59 genes by using Cytoscape software. The resulting layout of the network is presented in Figure 2A. 1906 nodes (proteins) and 2385 edges (interactions between proteins) composed the network (Table 4A – full list of nodes).

As a result, we were able to obtain the largest number of putative proteins that could be associated with the fibrogenic process (passing from 59 to 1906 proteins of interest), probably losing specificity, which will be improved in the subsequent steps of the strategy, using Venn's diagrams. The 1906 nodes were mapped to their UniProtKB identifiers obtaining 1879 reviewed proteins, to be used in further analysis (Figure 3A and Table 4). The remaining 27 nodes were chemical molecules, RNA species (miRNAs, piRNAs, etc), or protein complexes not mapped and reviewed in UniProt.

2.2 Data collection and identification of protein-coding genes with elevated expression in healthy liver and differentially expressed in hepatic fibrosis

To identify the protein-encoding genes showing high expression in healthy liver tissue, we used the transcriptomic datasets, published by Uhlén M. et al. [8]. We filter the entire transcriptome dataset obtaining 342 protein-encoding genes elevated in normal liver tissue. The total elevated protein-coding genes were constituted by four different categories (according to the criteria established by Uhlén M. et al. based on transcript expression levels), obtaining 135 tissue enriched, 49 groups enriched, 62 enhanced, and 95 mixed genes, respectively (see Table 1A and Table 5A). Once again, we matched these genes to their UniProtKB identifiers obtaining 341 reviewed proteins.

Concerning transcriptomics of the fibrotic liver, we exploited the RNA-seq dataset published by Hotta K. et al. [3] in which whole transcriptome shotgun sequencing (WTSS) was performed on liver specimens obtained from patients classified as having mild (fibrosis stages 0-2, n=39) or advanced (fibrosis stages 3-4, n=21) NAFLD. The fibrosis stage was classified according to Brunt [15]. A total of 1777 protein-encoding genes were reported as differentially expressed between mild and advanced NAFLD. All these genes were matched to their corresponding UniProtKB IDs, identifying 1721 reviewed proteins and used in the subsequent analysis (Table 1A and Table 6A).

2.3 Data collection and identification of secreted, plasma, and housekeeping protein-coding genes of the human proteome

Continuing with our analysis, we overlapped datasets regarding secreted protein-coding genes from three different publicly databases: Human Protein Atlas Portal [16], VerSeDa [17], and MetazSecKB [18]. The total number of protein-coding genes obtained from each database is summarized in Table 2A and the complete list of proteins with their respective UniProtKB identifier

in Table 7A. As depictured in figure 3A-a, from a total of 3.240 unique protein-coding genes identified as secreted proteins, 589 of them were common among datasets.

Plasma protein data from the three following different data resources: plasma proteome database [6] [19], the build 2017-04 of human plasma PeptideAtlas [7] [20], and human protein atlas portal [21], were collected to have a representative human plasma proteome. Eleven thousand two hundred eighty-two (11,282) unique total proteins were identified in plasma and mapped to their respective UniProtKB identifiers. As shown in Venn diagrams of figure 3A-b, 1.893 proteins were common among datasets. As in the former cases, proteins were mapped to their UniProtKB IDs for further analysis (Table 8A).

To retrieve data regarding the housekeeping proteome, we used the information about housekeeping protein-coding genes from the transcriptomic review published by Uhlén M. et al. [8]. A total of 7.579 protein-coding genes were detected by RNA-seq, as expressed in all tissues, and expected to be almost the complete human housekeeping proteome (Table 2A). They were classified as housekeeping protein-coding genes and mapped to their reviewed protein IDs, using the UniProt database (Table 9A).

2.4 In silico strategies for selection of candidates useful in liver fibrosis diagnosis

To identify the most interesting candidates involved in the fibrotic process, we overlapped the components of the PPI network for myofibroblast (with the total protein-coding genes showing elevated expression in normal liver (n=341) and those differentially expressed in the fibrotic liver (n=1721) (Figure 2A, Tables 4A-6A). We identified 210 interested proteins to be used in subsequent analysis (Figure 3A-b).

To complete our *in silico* strategy and because we were interested in proteins that can be measured in plasma, we combined the previously selected proteins with the datasets corresponding to secreted, housekeeping, and plasma proteins. Moreover, during the analysis, we applied two

different approaches designed as "Minimal and Maximal". Firstly, in the minimal process, we combine the selected proteins with the dataset of common proteins generated through the intersection of the secretome (n=589) and the plasma proteome (n=1893) datasets (Figure 3A-a,b and Figure 4A-a). Secondly, for the maximal approach, the interested candidates were merged with the dataset resulting from the union of the secretome (n=2939) and plasma proteome (n=11282) datasets (Figure 3A-a,b and Figure 4A-b). The dataset for housekeeping protein-coding genes was the same in both approaches (Table 2A).

When the individuated 210 candidate proteins were subjected to the minimal and maximal approach; Venn diagrams demonstrated that 9 and 35 protein candidates fulfill the selection criteria using the minimal and maximal method, respectively (figure 4A- a, b and tables 10A and 11A). Moreover, the following 9 candidates were common to both approaches: Insulin-like growth factor-binding protein 5, collagen alpha-3(VI) chain, collagen alpha-1(III) chain, EGF-containing fibulin-like extracellular matrix protein 1, ficolin-2, complement factor I, angiogenin, alpha-1-acid glycoprotein 2 and properdin. For a complete list of the individuated candidates, see Table 12A. Additional candidates were identified when considering the candidates as present in plasma but not reported as secreted proteins (see Table 12A)

2.5 Over-represented biological pathways for the selected protein candidates

Table 13A lists the first twenty significantly enriched biological processes, according to Reactome curated pathway database, in which the 35 protein candidates were involved. The most relevant pathways include as expected: extracellular matrix organization, degradation, assembly of collagen fibrils, regulation of Insulin-like Growth Factor (IGF) transport and uptake by Insulin-like Growth Factor Binding Proteins (IGFBPs), integrin and non-integrin membrane-ECM interactions, among others, representing mainly the liver fibrosis evolving process.

3. *References*

[1] Uhlén M, Fagerberg L, Hallström BM, Lindskog C, Oksvold P, Mardinoglu A, et al. Proteomics. Tissue-based map of the human proteome. Science 2015;347:1260419. https://doi.org/10.1126/science.1260419.
[2] Keen JC, Moore HM. The Genotype-Tissue Expression (GTEx) Project: Linking Clinical Data with Molecular Analysis to Advance Personalized Medicine. J Pers Med 2015;5:22–9. https://doi.org/10.3390/jpm5010022.
[3] Hotta K, Kikuchi M, Kitamoto T, Kitamoto A, Ogawa Y, Honda Y, et al. identification of core gene networks and hub genes associated with progression of non-alcoholic fatty liver disease by RNA sequencing. Hepatol Res Off J Jpn Soc Hepatol 2017. https://doi.org/10.1111/hepr.12877.
[4] Boers W, Aarrass S, Linthorst C, Pinzani M, Elferink RO, Bosma P. Transcriptional profiling reveals novel markers of liver fibrogenesis: gremlin and insulin-like growth factor-binding proteins. J Biol Chem 2006;281:16289–95. https://doi.org/10.1074/jbc.M600711200.
[5] El Taghdouini A, Sørensen AL, Reiner AH, Coll M, Verhulst S, Mannaerts I, et al. Genome-wide analysis of DNA methylation and gene expression patterns in purified, uncultured human liver cells and activated hepatic stellate cells. Oncotarget 2015;6:26729–45.
[6] Nanjappa V, Thomas JK, Marimuthu A, Muthusamy B, Radhakrishnan A, Sharma R, et al. Plasma Proteome Database as a resource for proteomics research: 2014 update. Nucleic Acids Res 2014;42:D959-965. https://doi.org/10.1093/nar/gkt1251.
[7] Schwenk JM, Omenn GS, Sun Z, Campbell DS, Baker MS, Overall CM, et al. The Human Plasma Proteome Draft of 2017: Building on the Human Plasma PeptideAtlas from Mass Spectrometry and Complementary Assays. J Proteome Res 2017. https://doi.org/10.1021/acs.jproteome.7b00467.
[8] Uhlén M, Hallström BM, Lindskog C, Mardinoglu A, Pontén F, Nielsen J. Transcriptomics resources of human tissues and organs. Mol Syst Biol 2016;12:862.
[9] The UniProt Consortium. UniProt: the universal protein knowledgebase. Nucleic Acids Res 2017;45:D158–69. https://doi.org/10.1093/nar/gkw1099.
[10] Wein SP, Côté RG, Dumousseau M, Reisinger F, Hermjakob H, Vizcaíno JA. Improvements in the Protein Identifier Cross-Reference service. Nucleic Acids Res 2012;40:W276-280. https://doi.org/10.1093/nar/gks338.
[11] Orchard S, Kerrien S, Abbani S, Aranda B, Bhate J, Bidwell S, et al. Protein interaction data curation: the International Molecular Exchange (IMEx) consortium. Nat Methods 2012;9:345–50. https://doi.org/10.1038/nmeth.1931.
[12] Fabregat A, Jupe S, Matthews L, Sidiropoulos K, Gillespie M, Garapati P, et al. The Reactome Pathway Knowledgebase. Nucleic Acids Res 2018;46:D649–55. https://doi.org/10.1093/nar/gkx1132.
[13] Hinz B, Phan SH, Thannickal VJ, Galli A, Bochaton-Piallat M-L, Gabbiani G. The Myofibroblast. Am J Pathol 2007;170:1807–16. https://doi.org/10.2353/ajpath.2007.070112.
[14] Kisseleva T. The Origin of Fibrogenic Myofibroblasts in Fibrotic Liver. Hepatol Baltim Md 2017;65:1039–43. https://doi.org/10.1002/hep.28948.
[15] Brunt EM. Nonalcoholic steatohepatitis: definition and pathology. Semin Liver Dis 2001;21:3–16.
[16] The Human Protein Atlas n.d. https://www.proteinatlas.org/ (accessed May 24, 2018).
[17] Cortazar AR, Oguiza JA, Aransay AM, Lavín JL. VerSeDa: vertebrate secretome database. Database 2017;2017. https://doi.org/10.1093/database/baw171.
[18] Meinken J, Walker G, Cooper CR, Min XJ. MetazSecKB: the human and animal secretome and subcellular proteome knowledgebase. Database J Biol Databases Curation 2015;2015. https://doi.org/10.1093/database/bav077.
[19] Plasma Proteome Database n.d. http://www.plasmaproteomedatabase.org/ (accessed July 12, 2018).

[20] PeptideAtlas n.d. https://db.systemsbiology.net/sbeams/cgi/PeptideAtlas/Search (accessed July 12, 2018).
[21] Search: protein_class:Plasma proteins - The Human Protein Atlas n.d. https://www.proteinatlas.org/search/protein_class:Plasma%20proteins (accessed July 13, 2018).

Figure 1: Venn diagrams showing the strategies for the individuation of protein-coding genes involved in myofibroblasts phenotype acquisition.

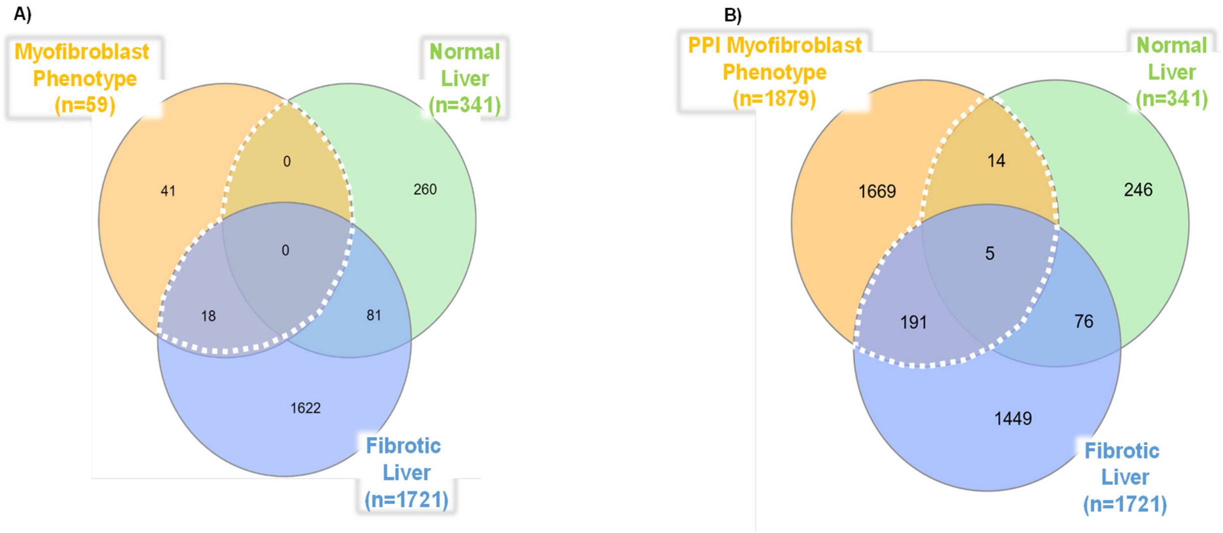

Figure 1A. a) Overlap between protein-codign genes classified as: presenting elevated expression in normal liver (light green), differentially expressed in fibrotic liver (light blue) and human transdifferentiated myofibroblast (orange), respectively. b) Overlap between the components of the protein-protein interaction biological network for human transdifferentiated myofibroblast and the protein-coding genes classified as elevated in normal and those differentially expressed in fibrosis (fibrotic liver).

Figure 2A: Layout of PPI biological network for transdifferentiated myofibroblast obtained with Cytoscape software.

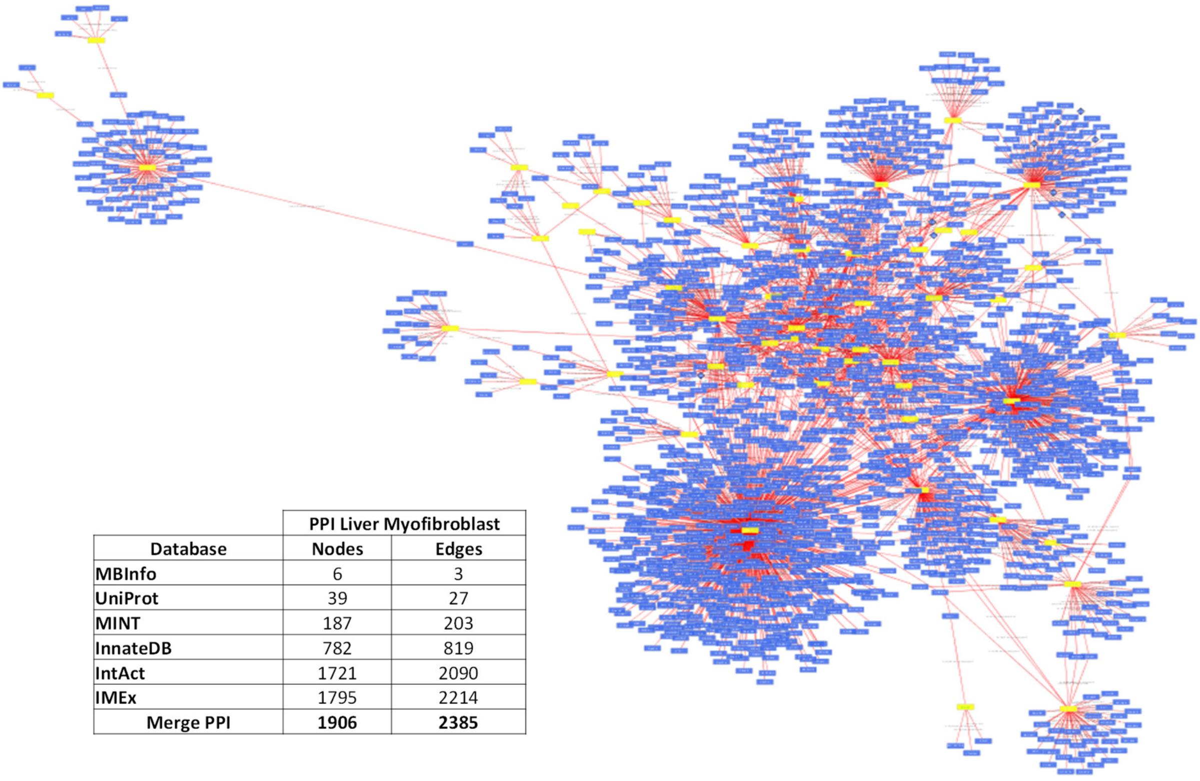

	PPI Liver Myofibroblast	
Database	Nodes	Edges
MBInfo	6	3
UniProt	39	27
MINT	187	203
InnateDB	782	819
IntAct	1721	2090
IMEx	1795	2214
Merge PPI	**1906**	**2385**

Figure 2A. Protein-protein interaction biological (PPI) network of the 59 identified proteins-coding genes strongly associated with the acquisition and maintaining of the myofibroblast phenotype for hepatic stellate cells. 59 central nodes (highlighted in yellow) generated a PPI biological network containing 1906 nodes and 2385 edges. PPI was created using Cytoscape software, the integrated network was filter by Human taxa and duplicated and self-loops were eliminated. Acquisition of PPI data was on May 2018.

Figure 3A. Venn diagrams depicting the overlap between datasets from different databases.

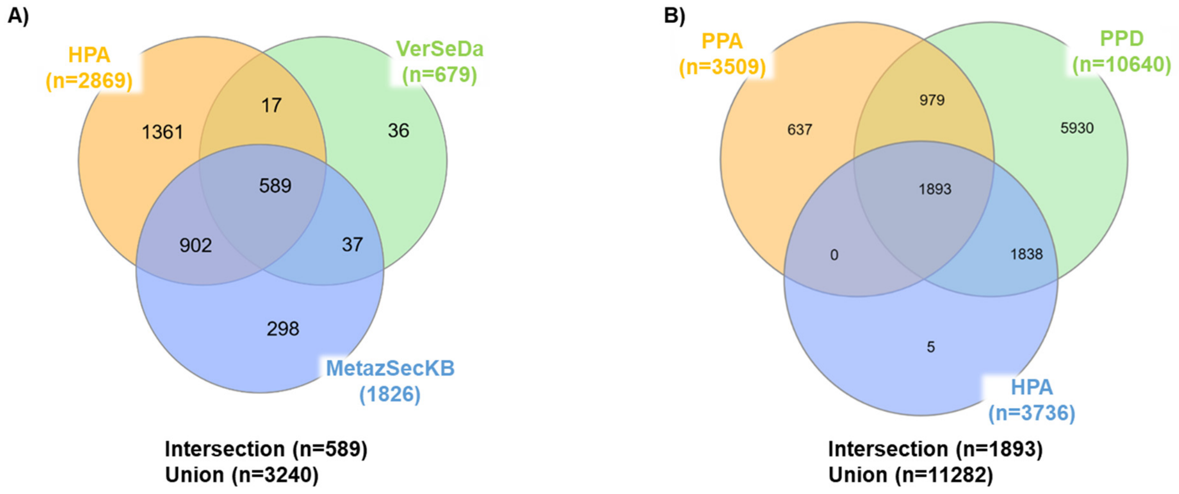

Figure 3A. The overlap between datasets for secretome and plasma proteome from different public databases. a) Human secreted proteins. Human Protein Atlas (HPA), Vertebrate Secretome Database (VerSeDa), Metazoa (human & animal) protein subcellular location, Secretome and subcellular proteome database (MetazSecKB). b) Human plasma proteome. Plasma PeptideAtlas (PPA), Plasma Proteome Database (PPD) and Human Protein Atlas (HPA).

Figure 4A. Minimal and maximal in silico strategies used in the identification of fibrosis-relevant protein-coding genes.

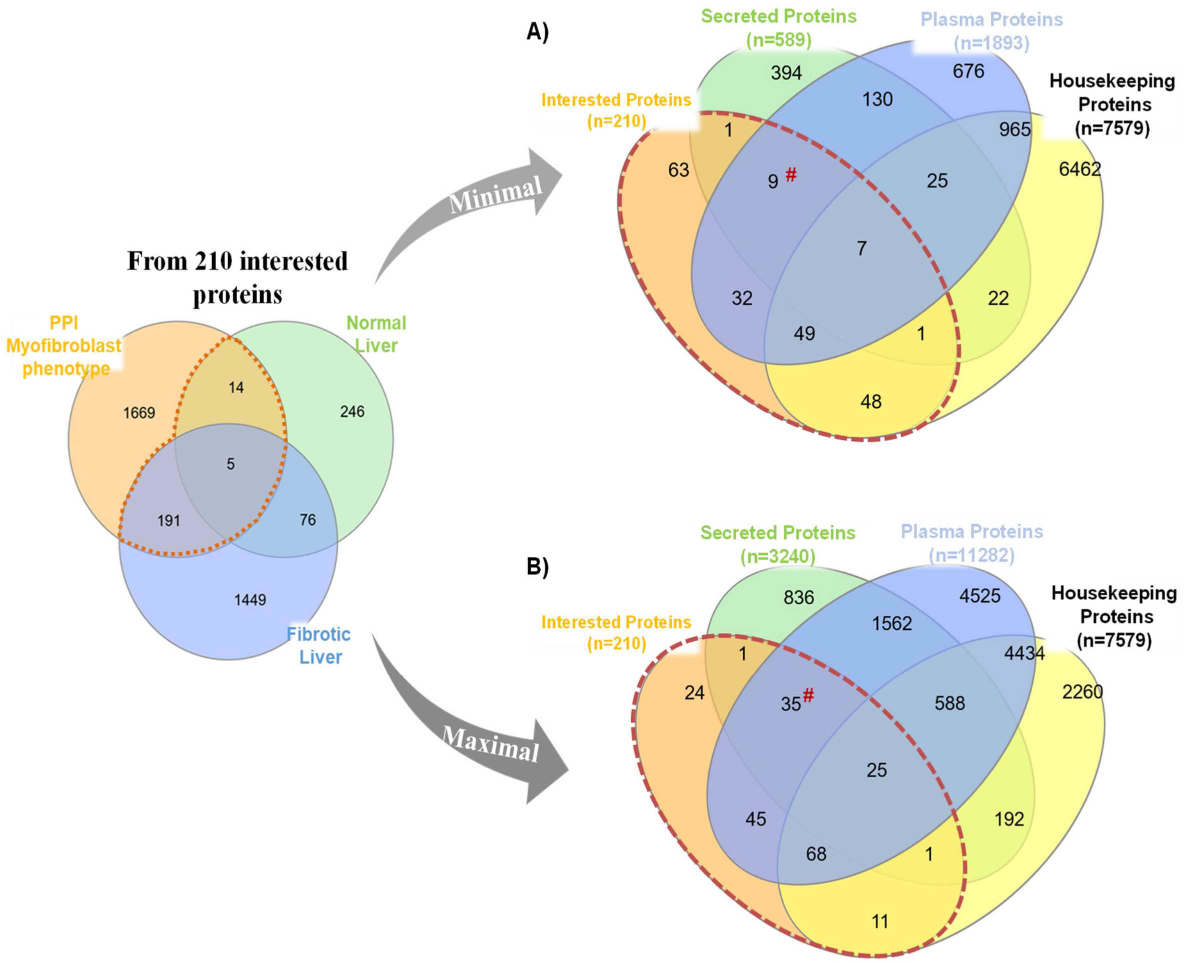

Figure 4A. Venn diagrams showing the *In silico* strategies used in the identification of fibrosis-relevant protein-coding genes. A) Minimal and B) Maximal strategies.
